# The Social Horizon

**Published:** 1893-04-01

**Authors:** 


### A Study in Modern Socialism.

### "THE SOCIAL HORIZON."

In our issue of December 31st last appeared an exhaustive criticism of Mr. Millin's book, "The Social Horizon," its aims, and tendencies. The author wrote us the following letter on the subject, which we submitted to the writer of the review in question. We also publish the critic's rejoinder :—

The notice of "The Social Horizon" in The Hospital of the 31st ult. is certainly one of the ablest of any that have appeared, and I only regret that it comes too late to be dealt with in "A Chapter with the Critics," which has gone to press for a second edition of the book. It is a curious and very significant fact that not a single reviewer has made the slightest attempt to deal with the string of assertions I made in the opening chapter of the book with regard to the aggrandisement of business and the tendency to monopoly. If you can explain that away, or show that in some way or other it may by-and-by be checked, you will have offered an effective criticism of the book. If you cannot do so you may argue as you will against Socialism, but facts are all against you. I was tickled at the way in which you point out the improvement in the circumstances of the working classes, and then immediately find yourself compelled to own that it is due to trades unionism. But trades unionism is to all intents and purposes Socialism within a circumscribed area. You have only to extend that area until it includes the whole population—and movements are all in that direction—and you have full-blown Socialism, modified, of course, so as to promote the welfare of the whole community. "It is generally admitted that railways, &c., may conveniently be worked by the State." Twenty years ago hardly anybody admitted it. "Money would not be forthcoming to run any industry at a loss, and if it was the taxpayer would know the reason why." Pretty nearly if not quite all the letters carried to every village in the land are carried at a loss ; a vast proportion of the parcels and most of the sixpenny telegrams. The State does find the money, and the taxpayer is very well satisfied with the reason why—it promotes the general convenience and comfort and welfare, and, taking the thing all round, it pays. Just so it will be in twenty years' time with a great many other things.

### The Critic's Rejoinder.

Mr. Froude showed us long ago that political history could be read in many ways, and that his keen eye could see in a monarch who had been held up to the reprobation of centuries a kindly and generous man, of the highest religious principles and the purest moral feelings, who had the unparalleled bad luck to have six bad wives in succession. But we are not all convinced by Mr. Froude. Similarly the author of "The Social Horizon" draws new deductions from the history of economics. Because he sees large industries take the place of small ones, because the large store occupies the space and takes over the business of a score of little shops, because one man manages things on a large scale instead of many managing them on a small one, he says that Socialism is coming and ought to come. None of his critics have controverted his facts ; therefore he declares that consciously or unconsciously, *bon gré mal gré*, all his critics agree with the deduction he makes from them. But one may admit the facts without accepting the inference. Our economic Froude is original in his view, but we are not convinced by it. The general opinion is that these great industries are the outcome and the triumph of the individualist system. On this ground you will find Socialists condemn them, protesting that whereas in the good old days the handicraftsman put heart and brain in his work, making it the reflex of his own individuality, heart and brain are now eliminated from his mechanical toil, and he is, literally, as in common phrase, only a "hand." In every large enterprise you will find that the energy, the genius of one man or one family has been the mainspring of its success. Give all the honour that is due to those who have worked under this man's commands as being faithful to their trust and capable in their degree ; it does not follow that any one among them could take his place. *Tel brille au second rang qui se perd au premier*, says the shrewd French proverb ; and in every large shop or factory you will find managers of departments who are invaluable where they are, but who, lacking that business prescience which is the essence of the successful man's nature, end in lean bankruptcy when they set up for themselves. Nation of shopkeepers though we are, we do not realise that genius differentiates one business man from another as much as genius differentiated Tennyson from the rhymer of the provincial newspaper.

Yet co-operative stores succeed, it may be urged. They do, especially if they have good managers and the shareholders leave these a free hand. The co-operation serves more to give the manager a sufficiency of capital to "run" his business in the most profitable manner than to contribute co-operatively to its successful conduct. As for the different results in the same business of joint stock and individual possession, let me give an instance from my own knowledge. Within a few miles of my house are two large steel works, one owned by a company, the other by three brothers. The company has not paid a dividend for several years, strikes and lock-outs alternate with each other ; men are taken on or sent off at every slight variation of the market, and a moderately long depression of the steel trade will probably result in the whole business being shut up. The other—the individualist—concern is as profitable as the joint-stock is the reverse. As the partners are not bound to disclose their profits, I would be rash to repeat the figures I have heard, but undeniably the business affords not only a handsome income to the proprietors, but steady work and good pay to those whom they employ. How is this ? Because of many things wherein the individual mind that has to ask leave of none can dare what the manager who has to keep down expenses for the sake of the balance-sheet cannot venture on. For example : If a new machine comes out which promises a saving in the cost of production, the individualist master will buy it, regardless of its initial price ; but let the joint-stock manager do so, and shareholders, who know very little of the business beyond the fact that they have money in it, will cry out, "Did we not pay so many thousands for plant not long ago ? The purchase of a new machine before the old is worn out is sheer extravagance"—and the energetic manager will stand a chance of being dismissed for his pains.

Other instances might be adduced, but this lay convenient to my hand, therefore I have given it.* And, in face of these facts, I venture to hold with the older economists, and to differ from the author of "The Social Horizon" in his opinion that large industries are socialistic in their tendency, and that their success depends in any degree upon socialistic methods in their management.

I see, too, that the author has assumed something which I never meant from my remarks on the effect of

* The report of the Massachusetts Labour Bureau gives some valuable statistics on this point. Private establishments, according to these, pay better in every way. The average wages per head in these was 474·37 dollars a year, and in joint-stock establishments 383·47, while the produce per dollar of capital invested was 2·58 dollars' worth in the private and 1.37 dollars' worth in the joint-stock. (Rae, "Contemporary Socialism" 418.)

trades unions. I said "trades unions have forced him (the employer) to give more and more to his workers, till the limit of wages is no longer fixed at the least the workman can afford to take, but at the utmost an employer can afford to give." This "utmost" may be much or little; that depends on the nature of the business. I made it very clear that trades unions had not in any way made plenty possible for the working classes; the plenty was there through economic conditions of a different class, and trades unions simply encouraged them to demand a larger share of it. Trades unions, as Mr. Chamberlain says, can hasten an inevitable rise or retard an inevitable fall in wages. This is what they can do; when they attempt to do more—to keep up wages permanently in face of a falling market—they enter upon a suicidal policy. They break down the wage fund upon which they must draw in the future; in the end they destroy the industry by which they hope to live. In short, they kill the goose that lays the golden eggs.

On this point I may have failed to define clearly the limits of what I meant as to the effect of the action of trades unions upon wages; but I never said, never implied, never thought for an instant that I could seem to imply that "trades-unionism is to all intents and purposes socialism within a circumscribed area." For one thing, the phrase "Socialism within a circumscribed area," is in itself contradictory. "Society" cannot be limited to the lower thirty millions any more than to the upper ten thousand, and a socialistic scheme which leaves out any body—yes, even an unhappy duke or a benighted millionaire—is to that extent imperfect. Indeed, the weak point of all the Socialist schemes I have ever heard of seems just to be that they can be successfully carried out only by the few, the self-sacrificing idealists, not by the all and sundry of humanity. On this point I shall have something to say farther on; at present I wish to express as emphatically as I can the conviction, shared by wiser thinkers and keener observers than myself, that trades unions are inspired by a strictly individualistic spirit. They are not even an organisation of one class against another, of labourers against capitalists, but an attack by certain sections of labour on the wage fund upon which all sections must draw. Paul's increased wage is taken from Peter's pocket. For example, a builder undertakes to erect a house for a given sum, but the bricklayers insist on receiving higher wages than his estimate allows. If so, he will certainly try to diminish the wages of carpenters, plumbers, glaziers, all the other workmen employed on the house in order to recoup himself. If, however, the other workmen follow the bricklayers' example, and the employer is forced to yield to their demands, with the result that he himself has no profit from the work, he will certainly give up an employment so unremunerative. In fact he must, for he has no capital wherewith to undertake another venture. The wage fund has been destroyed; the industry is crippled. This excess of trades-unionism has been in the end suicidal; and will anyone pretend that these bricklayers, carpenters, plumbers, and the rest had any social end in view? They sought to fill their individual pockets; and if the builder were ruined thereby, or were forced to make up for what they got by asking higher rents and prices for the houses he built—the cottage of the workman as well as the mansion of the capitalist—that, they considered, was no concern of theirs.

Yet, in the intricate relations of society it was their affair, and the end of it all may be that what they pocket in wages they have to pay out in house rent. This, however, is beside the mark at which I was aiming, which was simply to show that the ideal of trades-unionism was not socialistic, but the reverse. Upon the good or evil of this ideal I offer no opinion. But at least it will be admitted that trades-unionism is in no way a product of socialism. Trades unions are the natural development of the craft guilds of the middle ages, and they have pursued much of the same course. Originated to redress a grievance or facilitate the development of an industry, they often ended by attempting to create a monopoly in it. Some of our trades unions are doing the same. The Boiler Makers' Union, to which a large number of the workers on iron ships belong, has recently issued a mandate limiting the number of apprentices to be taken on in each ship-building yard. The intention is, of course, to keep up wages by limiting the supply of labour—a purely individualist ideal. But the end may be that, if there is not labour enough at command in this country to build as many ships as are required, the ship-building industry will be largely driven to other nations—for, after all, we are not the only ship-builders in the universe—and work and wages be no more plentiful than they are now.

In another way the action of trades unions threatens to injure the interests it intends to serve. Said a master printer the other day: "I pay all my compositors the same wage, that which the society commands. Some of the men are worth a great deal more to me than I pay them; some are not worth half as much; but I'm bound to treat them all alike. The only advantage a good workman has over a bad one is that when work is scarce he is kept on when the other is dismissed." So if this be socialism it is the socialism that sacrifices the best to the worst, the moral result of which is apt to be that the good worker, knowing that he cannot "better himself," becomes indifferent, and the bad worker, knowing that the union has secured his wage for him, becomes more careless than before. Here the trades union has forced the employer to give all he can afford, but it prevents his dividing that sum according to the merits of the workers. Indeed, it may be doubted if in these days trades unions, in so far as their influence on wages are concerned, are much needed. The competition among employers for labour is a surer means of keeping wages up than all the unions in the world, just as that same competition raised the price of labour in spite of all legislative enactments to keep it down in the generation after the population of England had been decimated by the "black death," and workmen were few.

So much for trade unions and their supposed socialistic constitution. On another point the author thinks he has tripped up his Hospital critic. He says: "Pretty nearly if not quite all the letters carried to every village in the land are carried at a loss, and vast proportions of the parcels and most of the sixpenny telegrams. The State does find the money, and the taxpayer is very well satisfied with the reason why—it promotes the general convenience and comfort and welfare, and, taking the thing all round, it pays." He thinks that this controverts my statement that "money would not be forth-coming to run any industry at a loss." Yet it does not seem to me that he shows that the Post Office conducts its business at a loss; "taking the thing all round it pays," says he. There is nothing socialistic in that sentiment; it is a principle applied in the most individualistic concerns. Get into confidential discourse with a draper, and he will confide to you that he loses annually on his dressmaking department; he has to pay his cutter and fitter high wages; he has to keep a larger staff of workers than he can fully employ all the year round, because in the busy season ladies are so impatient to have their gowns. Why, then, does he not lop off the unprofitable branch? Because he knows that the reputation of his good dressmakers brings women to buy their gowns at his shop, and he makes a profit out of the sale of materials more than sufficient to cover the loss in the other department. "Taking the thing all round, it pays." Again, taking Post Office work as an example, we find a case very much to the point. A certain steamship company has a contract with the Government to carry the mails to islands in the West of Scotland. Even in summer some of their steamers are run at a loss; in winter, it is probable, nearly all. Why does the company take such unprofitable business? Not, we may be sure, because of a disinterested desire for the convenience and comfort of the dwellers in remote and barren islands. Not even because the Government payment is so large as on the whole to be remunerative, but because if this company did not accept the contract some other firm would, which might then be able to rob the first company of some share of its very profitable goods, and, on some routes, summer passenger traffic. Is it not said that in our railways first-class passengers are conveyed at less than the cost of transit, while the deficit they cause is made up by the more numerous and less-exacting third-class travellers, and still more by cattle and minerals? In every trade a direct loss is tolerated if, through the convenience it affords, it leads to indirect profit, and, "taking the thing all round, it pays."

So far I have dealt only with the author's review of my review. In face of the inferences he had drawn from my words—inferences which others as well as myself will, I think, hold to be somewhat remarkable, I felt myself justified in explaining at some length what I really did mean. But I would fain ask him if indeed it is a Socialistic commonwealth that he sees predicted—since he makes himself an augur—in the hecatombs of the wholesale meat trade? Is it not rather the individualist principle on a larger scale? Even State interference does not inevitably mean Socialism. Socialism was neither the intention nor the result when the State meddled with religious opinion by means of stake and gallows. Their is no Socialism in the existence of factory inspectors, any more than in the existence of gaols and judges. That implies only such protection of life and property as an individual may justly claim from the central authority. Because the word Socialism is on every lip just now, we are apt to think that every change in law or custom has a Socialist tendency, but if he were examined by, say, the spirits of Marx and Lassalle, we doubt if the author of "The Social Horizon" could pass as a true Socialist.

The fundamental idea of Socialism is the destruction of private property. "From all according to their powers to all according to their needs," is the popular formula, which forgets the fact that perverse human nature can wonderfully stretch its needs and limit its powers when some such vulgar force as hunger does not exist to keep the two in balance. The question is, will progress and productivity go on if the incitement of personal gain be removed? The answer to this must be sought in such social experiments as have been already made. For various reasons these have been most numerous in the United States. Forty-two societies have been founded on purely Socialist principles, and have failed. We all know something of the Brook Farm experiment; why did not that succeed when it was formed by such pure souls, actuated by such noble motives? Because, as Horace Greeley epigrammatically put it, there came with these "scores of whom the world was quite worthy, the conceited, the crotchety, the selfish, the headstrong, the pugnacious, the unappreciated, the played-out, the idle, the good-for-nothing generally, who, finding themselves utterly out of place, and at a discount in the world as it is, rashly conclude that they are exactly fitted for the world as it ought to be." W. H. Channing, who had been a member of the community, said of it, "The great evil, the radical practical danger, seemed to be a willingness to do work half thorough, to rest in poor results, to be content amid comparatively squalid conditions, and to form habits of indolence." In all the other communities the result has been the same. Idleness and bad management have led to ruin.*

But there are seventy-two communities which have been economically successful. These are all religious bodies, such as the Shakers and Rappists, and have therefore an ideal different from the material one of our Socialist preachers. They regard idleness as a sin, second only in heinousness to disobedience to elders; and the elders have the power not only to make the idler uncomfortable, but in the last resort to dismiss him. Moreover, as celibacy is one of their laws, the community consists entirely of adult workers, and has not the burden of supporting children in their helpless years. Yet, even here, with everything to make for prosperity, the condition of the members is poorer than that of outside workers, and they own that an outsider can do nearly twice as much in a day. Thus, though prosperous, these communities are not so prosperous as, with such good economic conditions, one would expect.

If communities which thus have every weight lifted from them cannot equal, much less surpass, in productivity and success the work of the outside individualist world, what hope is there for any scheme of national Socialism where idlers could not be dismissed at will, and where the community would have to support and educate children until they reached an age to contribute to its resources? Our present development, faulty and imperfect as it is, is proceeding on natural lines. It develops individual energy by the hope each man can cherish of improving his condition. I, who am no Socialist, would gladly see the opportunities of all made more nearly equal, and it seems to me that by co-operation, education, the development of new outlets for capital and industry, and the opening up of communication between remote districts of the country, much may be, and is being, done, to bring this about. But let each man have the right to his own earnings; let him have the right to bequeath his savings to those whom he loves; let neither State help nor State confiscation deprive him of the inspiring sense of individual responsibility, without which he has little chance of being either a good worker, a good parent, or a good citizen.

### The Author's Reply.

It was, of course, my own fault that you should have received as for publication a letter which I wrote hurriedly under the pressure of other engagements, and which I intended merely as a private communication to yourself. As, however, by your courtesy, I have been permitted to see your comments before publication, I have, of course, no fault to find, and, indeed, I cannot but feel honoured by the attention of which you have deemed this book of mine worthy. Nevertheless, you have taken me somewhat unawares, and on that ground I have asked your permission to say a few words in reply.

Had I been writing for the general readers of The Hospital, I should certainly not have made so crude a statement as that you have only to extend trade unionism, and you have full-blown Socialism, without some amplification and elucidation of my meaning. I must let that and many other points pass however. All I can reasonably ask you to afford me space for is to re-assert the main proposition of my book which, in your own language, is "that large industries are socialistic in their tendencies."

There are four links in my chain of reasoning, and unless you can break one of these links you may assert your dislike to Socialism, you may set forth any number of proofs that it has failed in the past and is likely to fail in the future, and you may pass any strictures you please on the follies and failings of human nature, but you will not have affected my conclusions in the least. In the first place I appeal to facts within the knowledge of everybody in evidence of the all but universal tendency for small businesses to give place to large

* See "Rae's Contemporary Socialism," p.p. 402, 403.

ones, and for large ones to amalgamate. In the next place I assert that this drift has been observable all through our recent history, at least; that it is stronger now than it ever was; and that, as we are only on the threshold of the age of electricity, that tendency is likely to be indefinitely accelerated in the future. Thirdly, I point out—what, indeed, is perfectly self-evident—that if this drift continues uninterrupted, monopolies will become general. Lastly, I contend that it will be impossible for the community to tolerate monopolies, and that it will sooner or later be bound in self-defence to step in and assume control, just as London is about to do in the matter of water supply and public vehicles.

Now, sir, if you are prepared to say that there is no such tendency, or that if there is such a tendency it is not likely to continue, or that if it does continue it will not lead to monopoly, or that if it does lead to monopoly society is not likely to feel any inconvenience and will patiently submit to have most, if not all, the necessaries as well as the luxuries of life in the hands of monopolists—then, sir, you have answered my book. I respectfully assert that you have not successfully assailed one of these propositions, nor has any other reviewer done so. Notwithstanding your acute and able criticism, I still adhere most firmly to the belief that the keener and the more selfish and unscrupulous becomes competition, the more swiftly shall we move on towards monopolies; that by way of monopoly Socialism will be established; that Socialism, rightly understood, is order and beneficence; and I have not a doubt that towards this order and beneficence Providence is swiftly impelling us by all the forces of our day. Thanking you for your fair and courteous criticism of my book, and wishing I had more space in which to cross swords with you,—I am, yours very truly,

The Author of "The Social Horizon."

[It is true that co-operative stores and the principle they represent, have developed during the last decade. There is evidence, however, that the principle does not work to the satisfaction of an increasimg number of customers, and the further multiplication of these stores is doubtful, if not stayed. The experience of California and of the county of Kent especially prove that small holdings can be farmed at a profit, where bigger agricultural ventures fail. Further, a clearer knowledge of the dangers and the expenditure entailed by the purchase of the water supply of London, makes it more than probable that that supply will remain in the hands of the companies under Parliamentary regulation and not be bought up by the County Council. It is also very doubtful if the whole of the public vehicles of London will be acquired by the representatives of the ratepayers. The three foregoing links are therefore, to say the least, brittle, if some of them indeed have any existence at all in fact. We have abundant evidence to show, that whatever the tendency towards monopoly may have been in recent years, that tendency is disappearing, rather than increasing, owing to its monetary failure. To contend that a few members of the population elected by the ratepayers are competent to conduct every kind of business successfully to the advantage of the whole community, is too obviously ridiculous to be seriously put forward by any responsible thinker and statesman. Individually these representatives would not venture to put their own money into an undertaking so controlled, and any community foolish enough to try the experiment will soon be cured of its folly.—Ed., *T. H.*]

